# Characteristics and outcomes of doctors in a substance dependence monitoring programme in Canada: prospective descriptive study

**DOI:** 10.1136/bmj.a2098

**Published:** 2008-11-03

**Authors:** Joan M Brewster, I Michael Kaufmann, Sarah Hutchison, Cynthia MacWilliam

**Affiliations:** 1 Dalla Lana School of Public Health, University of Toronto, 155 College Street, Toronto, ON, M5T 3M7, Canada; 2 Ontario Physician Health Program, Ontario Medical Association, Toronto; 3 Ontario Medical Association, Toronto

## Abstract

**Objective** To describe the characteristics at enrolment and outcomes of doctors in a substance dependence monitoring programme in Canada.

**Design** Prospective descriptive study.

**Setting** Provincial physician health programme, Canada.

**Participants** All 100 doctors consecutively admitted to a substance dependence monitoring programme and followed until completion of monitoring or on leaving the programme.

**Main outcome measure** Relapse during long term monitoring for five years.

**Results** Ninety per cent of the doctors enrolled on the programme were men, 66% were married or living with a partner, 44% had had previous treatment for substance dependence, and 36% had had previous psychiatric treatment. Smokers were over-represented compared with the general population of US doctors (38% *v* 5%). During the monitoring period 71% of participants had no known relapse. An additional 14% went on to complete the programme, after some form of relapse. In total, 85% of the doctors successfully completed the programme.

**Conclusion** In this cohort of doctors enrolled on the Ontario Physician Health Program for substance dependence, most were men who were dependent on alcohol or opioids. Smokers were over-represented compared with the general population of US doctors. Eighty five per cent successfully completed the programme.

## Introduction

Programmes to help doctors who are dependent on substances such as drugs and alcohol exist in every US state^1^ and every Canadian province,^2^ in collaboration with medical associations or regulatory bodies. The British Medical Association also has developed a peer support programme.^3^ Descriptions of participants with drug related problems in physician health programmes consistently show that alcohol is the most common drug of choice, followed by opioids, and that multiple drug use is common.^4^ ^5^ ^6^ ^7^ Female doctors are generally under-represented as participants.^6^ ^7^ ^8^ Among these doctors, long term recovery rates are higher than those for substance use disorders in the general population, often exceeding 85%.^4^ ^8^ ^9^ ^10^

In Canada the Ontario Physician Health Program is among the largest of such programmes and, by 2002, had enrolled 100 doctors for monitoring recovery from substance dependence. Recovering doctors who choose monitoring are enrolled by a case manager from the programme after they have been diagnosed as dependent on substances, successfully treated, and established in early stable remission. Contract requirements for monitoring include visits to an addiction medicine doctor, visits to a family doctor for routine health needs, attendance at facilitated health professional support groups, attendance at mutual support groups in the community, monitoring in the workplace, and random screening of urine for alcohol and drugs of misuse. The usual agreement for monitoring is five years of continuing progress in recovery. Contingencies built into the monitoring agreement can result in details of the case being reported to the College of Physicians and Surgeons of Ontario, the regulatory body, when aspects of the contract are breached.

By the end of 2007 all of the first 100 doctors enrolled on the Ontario Physician Health Program for monitoring of substance dependence had completed contracts. We describe these doctors and their outcomes.

## Methods

Participants were the first 100 doctors enrolled in the Ontario Physician Health Program for monitoring of substance dependence after the formation of the programme in 1995. All were diagnosed as dependent on substances according to criteria of the *Diagnostic and Statistical Manual of Mental Disorders*, fourth edition, and treated for substance dependence, usually in a residential abstinence based programme for 4-6 weeks, before entering the monitoring programme.

### Intake measure and outcome measure (relapse)

On entering the monitoring programme, the doctors completed a clinical intake questionnaire detailing personal characteristics, history of substance misuse in the family, professional education and practice, licensure, history of substance use, treatment history, psychiatric history, and experience of physical and emotional abuse.

While being monitored by the physician health programme, any use of alcohol or an illicit drug or use of any psychoactive drug not prescribed by a participant’s doctor was considered to be relapse. Relapse ranges from single use to full resumption of dependence on substance use. After brief relapse, participants may continue the monitoring programme to successful completion; more serious relapse may lead to the doctor having to restart the programme under a new contract or leaving the programme.

### Data analysis

The data were analysed using SPSS version 15.0. We summarised the characteristics of participants using frequencies for categorical variables and means and standard deviations for continuous variables.

## Results

The table[Table tbl1] shows the personal characteristics of the participants, details of their practices, and drug and treatment histories on entry to the monitoring programme. In total, 90% of participants were men; two thirds (66%) were married or living with a partner. The mean (SD) age at enrolment was 45 (11) years, with a mean (SD) years experience of medical practice of 16 (11) years. Almost a half (48%) were in general practice or family practice. Most practised patient care in single or group practices, and most practised in urban areas, communities of 100 000 or more. These characteristics were generally similar to the population of Ontario doctors (Member Services Department, Ontario Medical Association). The licences of two thirds (69%) of participants were unaffected at the time of enrolment; the remainder (31%) were suspended or restricted (mostly to prevent the prescribing of narcotics). Two thirds of participants enrolled to satisfy a requirement of the College of Physicians and Surgeons of Ontario.

**Table tbl1:** Participants’ personal characteristics and history of drug use and treatment at enrolment in the Ontario Physician Health Program

Variable	No (%)
No of participants (n=100)	
Men	90 (90)
Women	10 (10)
Marital status (n=100):	
Married or living with a partner	66 (66)
Single	34 (34)
Specialty (n=92):	
General practice or family practice	47 (51)
Specialist	45 (49)
Practice setting (n=86):	
Single practice	35 (41)
Group practice	20 (23)
Other	31 (36)
Practice community size (n=94):	
<5000	3 (3)
5000-24 999	7 (7)
25 000-99 999	18 (19)
100 000-500 000	39 (41)
>500 000	27 (29)
Status of medical licence (n=96):	
Unaffected	66 (69)
Restricted or suspended	30 (31)
Involved with licensing body* at enrolment (n=99):	
Yes	65 (66)
No	34 (34)
Primary drug of choice (n=95):	
Alcohol	48 (51)
Opioids	35 (37)
Other	12 (13)
Previous treatment for drug related problem (n=97):	
Yes	43 (44)
No	54 (56)
Current tobacco smoker (n=93):	
Yes	35 (38)
No	58 (62)
Previous psychiatric treatment (past five years) (n=100):	
Yes	36 (36)
No	64 (64)
Ever experienced physical or emotional abuse (n=96):	
Yes	58 (60)
No	38 (40)
Ever been sued for malpractice (n=96):	
Yes	15 (16)
No	81 (84)
Mean (SD) age at enrolment (n=100)	45.4 (10.5)
Mean (SD) years in practice (n=94)	16.2 (10.8)

*College of Physicians and Surgeons of Ontario was aware of participant’s contract with physician health programme.

Half of the participants (51%) used alcohol as their primary drug of choice, one third opioids (37%), and the remainder (36%) other drugs such as benzodiazepines, stimulants, or illicit drugs such as cannabis or cocaine. Nearly half (44%) had received drug related treatment at some time before the most recent treatment. More than a third (38%) were current smokers, higher than the current rate of smoking among the general population of US doctors, which is about 5%.^11^ Thirty six per cent of the participants had had psychiatric treatment in the previous five years. More than half (60%) reported ever experiencing some form of physical or emotional abuse.

During the monitoring period 71% of participants had no known relapse. An additional 14% went on to complete the monitoring programme after some form of relapse. In total, 85% of the doctors successfully completed the programme.

## Discussion

Doctors who entered the Ontario Physician Health Program for substance dependence were typically middle aged, married men dependent on alcohol or opioids. Overall, 85% of the participants successfully completed the programme.

The strength of our study is that the participants were 100 doctors consecutively admitted for monitoring of substance dependence in a physician health programme, without selection. Results were accumulated over 10 years, the time taken for 100 doctors to complete at least five years of monitoring.

One possible weakness of the study is that the characteristics of doctors entering the programme could change over this time span. Measures on the intake questionnaire were by self report and subject to the limitations of that method. Possibly we failed to identify some relapses, leading to misclassification of outcomes. The relatively small number of participants prevented us from studying the relation between characteristics of the participants at intake and relapse.

This profile of the personal characteristics of the participants and their practices is typical of other reports of participants in physician health programmes, where women are under-represented.^4^ ^5^ ^6^ ^7^ ^8^ The high rate of smoking among the participants compared with the general population of US doctors (38% *v* 5%) and the high success rate is comparable to that of other North American physician health programmes that report outcomes for monitoring substance dependence.^4^ ^8^ ^9^ ^10^

Doctors entering a substance dependence monitoring programme are more likely to be men and to be smokers than other doctors. They are usually known to the licensing body. Their primary drugs of choice are alcohol or opioids. A structured monitoring programme after abstinence based primary treatment for these substance dependent doctors, with regulatory contingencies for lack of adherence, can produce successful results. Further research is needed to examine the relation of the characteristics of the doctors and components of the programme to relapse, so that monitoring can be tailored to the specific needs of participants.

**What is already known on this topic**

Studies have shown a high rate of successful outcomes in doctors enrolled in monitoring programmes for substance dependence

**What this study adds**

In a sample of 100 doctors consecutively admitted to a physician health programme, most were men and the drugs of dependence were alcohol or opioids
After abstinence based residential treatment, 85% of the doctors successfully completed the comprehensive programme of monitoring with contractual contingencies

## Notes

Contributors: JMB contributed to the design of the intake questionnaire and the study design, reviewed the literature, analysed the data, and led the writing of the paper. IMK designed the monitoring programme, contributed to the study design, and had a major role in writing the paper. CMacW administered the monitoring programme in later years, ensured accurate data collection and entry, and reviewed the paper. SH administered the monitoring programme early in the programme, ensured accurate data collection and entry, and reviewed the paper. JMB and IMK are the guarantors.

Ethical approval: This study was a secondary analysis of an anonymous clinical database. It was approved by the Health Sciences Research Ethics Board of the University of Toronto.

Provenance and peer review: Not commissioned; externally peer reviewed.

## Notes

**Cite this as: ***BMJ* 2008;337:a2098
